# Longitudinal trajectories of peer relations in children with specific language impairment

**DOI:** 10.1111/jcpp.12190

**Published:** 2014-01-11

**Authors:** Pearl L H Mok, Andrew Pickles, Kevin Durkin, Gina Conti-Ramsden

**Affiliations:** 1 The University of ManchesterManchester, UK; 2 King's College LondonLondon, UK; 3 University of StrathclydeGlasgow, UK

**Keywords:** Specific language impairment, peer relations, developmental trajectories, prosocial behaviour, pragmatic language impairment, autistic symptomatology

## Abstract

**Background:**

Peer relations is a vulnerable area of functioning in children with specific language impairment (SLI), but little is known about the developmental trajectories of individuals.

**Methods:**

Peer problems were investigated over a 9-year period (from 7 to 16 years of age) in 171 children with a history of SLI. Discrete factor growth modelling was used to chart developmental trajectories. Multinomial logistic regression analysis was conducted to investigate factors associated with group membership.

**Results:**

Four distinct developmental trajectories were identified: low-level/no problems in peer relations (22.2% of participants), childhood-limited problems (12.3%), childhood-onset persistent problems (39.2%) and adolescent-onset problems (26.3%). Risk of poor trajectories of peer relations was greater for those children with pragmatic language difficulties. Prosocial behaviour was the factor most strongly associated with trajectory group membership. Overall, the more prosocial children with better pragmatic language skills and lower levels of emotional problems had less difficulty in developing peer relations.

**Conclusions:**

Analysis of developmental trajectories enriches our understanding of social development. A sizeable minority in the present sample sustained positive relations through childhood and adolescence, and others overcame early difficulties to achieve low levels of problems by their early teens; the majority, however, showed childhood-onset persistent or adolescent-onset problems.

## Introduction

Many children presenting to clinical and psychological services manifest difficulties in peer relations. A common, although often overlooked, source of peer difficulties is language impairment. Communication is fundamental to the initiation and maintenance of successful peer relationships (Ladd, Kochenderfer-Ladd, Visconti & Ettekal 2012; Rubin, Begle & McDonald 2012), and a growing body of literature points to the presence of peer problems in children with specific language impairment (SLI). SLI is a common developmental disorder affecting 5–7% of children (Tomblin et al., 1997). Difficulties in expression and/or comprehension of language make participation in conversation challenging, with the consequence that children with SLI engage less in active interactions than do those with typical language, enter less frequently into positive social interactions, are less sensitive to the initiations offered by others, and manifest situationally inappropriate verbal responses (Craig & Washington 1993; Fujiki, Brinton, Isaacson & Summers 2001; McCormack, Harrison, McLeod & McAllister 2011; Vallance, Im & Cohen 1999). Experiencing difficulties with peer relations during childhood means that many children with SLI enter adolescence less equipped in the skills needed for this area of life.

SLI, however, is a heterogenous disorder with some children exhibiting particular difficulties with connected discourse, making inferences and language use. In the 1990s, these children tended to be referred to as having semantic/pragmatic difficulties but more recently, this term has been supplanted by the label pragmatic language impairment or PLI (Bishop, 1997; Botting & Conti-Ramsden, 2003). Furthermore, there are children with SLI who also exhibit autistic symptomatology (Conti-Ramsden, Simkin & Botting 2006; Loucas et al., 2008) and children with PLI who do not have features of ASD (Bishop & Norbury 2002). Thus, both pragmatic language abilities and autistic symptomatology may be implicated in the manifestation of difficulties in peer relations among SLI.

Conti-Ramsden and colleagues investigated the developmental trajectories of behavioural, emotional and social difficulties in individuals with a history of SLI from childhood to adolescence, and found that peer relations was the most developmentally vulnerable area of functioning (St Clair, Pickles, Durkin & Conti-Ramsden, 2011). The investigators observed an increase in peer problems and in the proportion of individuals functioning in the impaired range from childhood to adolescence. By 16 years of age, against national norms, nearly 40% of adolescents with SLI appeared impaired in their interactions with peers.

While the above study yields valuable information on the overall mean trajectory of peer relations over time, it does not elucidate possible individual differences. Identifying meaningful groups and developmental patterns is crucial towards helping understand the heterogeneity among children with a disorder and may also serve to inform strategic identification, referral and interventions (Bongers, Koot, van der Ende & Verhulst 2004; Conti-Ramsden, 2008; Nagin & Odgers 2010). To our knowledge, this study is the first to determine empirically group differences in the developmental course of peer relations amongst individuals with a history of SLI, examining changes in peer relations from childhood to adolescence. Although in general children with SLI are sociable (Wadman, Durkin & Conti-Ramsden, 2008), findings have been mixed with respect to prosocial behaviour. There is some evidence that, as a group, children with SLI are less likely to exhibit skilled prosocial behaviour (Fujiki, Brinton, Morgan & Hart 1999; Stevens & Bliss 1995) but Farmer (2000) found that 10-year-old children with SLI displayed levels of prosocial behaviours that were similar to their typically developing peers. Children with SLI also tend to be rated as more withdrawn than peers (Brinton & Fujiki 2002; Cohen, Barwick, Horodezky, Vallance & Im 1998; Fujiki et al., 2001; Redmond & Rice 1998) yet are at heightened risk of exhibiting externalizing problems and antisocial conduct disorders (Beitchman et al., 2001; Brownlie et al., 2004; Conti-Ramsden & Botting 2004).

We investigate longitudinal trajectories of peer relations over a nine year period in a sample of children with a history of SLI. We examine factors potentially influencing the trajectories, including expressive, receptive and pragmatic language skills, as well as emotional symptoms, conduct problems, hyperactivity and prosocial behaviour. We also investigate associations with autistic symptomatology.

## Method

### Participants

Participants have a history of SLI and were originally part of a wider study: the Manchester Language Study (Conti-Ramsden & Botting 1999; Conti-Ramsden, Crutchley & Botting 1997). The initial cohort of 242 children (6;6–7;9 years) was a random sample of 50% of all 7-year-olds attending 118 language units across England. Language units are specialized classes for children who have been identified with primary speech and language difficulties; the units are usually attached to mainstream schools. Children were excluded from the study if they were reported by their teachers as having frank neurological difficulties, hearing impairment, a diagnosis of autism or a general learning disability.

Participants were contacted again at ages 8 (*N* = 232), 11 (*N* = 200), 14 (*N* = 113) and 16 (*N* = 139). Ethical approval was obtained from The University of Manchester and written informed consent was gained from all participants at each stage. The attrition observed was partly due to funding constraints at follow-up stages of the study. Participants for follow-up stages of the study were retained on the basis of traceability and geographical accessibility. Measures of peer relations were available at ages 7, 8, 11 and 16. Only individuals who had peer measures for at least 3 of the 4 time points have been included: total of 171 children (25% girls). Their psycholinguistics profiles at 7 and 11 years of age are shown in Table 1. The average standard scores for receptive language at both ages and for expressive language at age 7 were around 1 SD below the population mean, whilst average expressive language score at age 11 was close to 2 SD below. The mean performance IQ (PIQ) scores fell between ages 7 and 11 (Conti-Ramsden, St Clair, Pickles & Durkin 2012). At age 7, PIQ was slightly above the population mean, and by age 11, it had fallen to 1 SD below. There was no difference in the receptive language, expressive language and PIQ standard scores at age 7 between those who participated at age 16 and those who did not, *p*s > .3. Further demographics information of the participants is available as supplementary online materials.

**Table 1:** Mean (SD) of language and PIQ scores of children at ages 7 and 11

	Age 7	Age 11
Receptive language standard scores^a^	83.7 (11.2)	86.6 (15.5)
Expressive language standard scores^b^	83.2 (10.0)	73.6 (11.7)
PIQ standard scores^c^	105.7 (14.9)	85.6 (23.5)
*N*	168	169

a Receptive language measures at ages 7 and 11: Test for Reception of Grammar (Bishop, 1982).

b Expressive language measures: age 7 – Bus Story Test (Renfrew, 1991); age 11 – Recalling Sentences subtest of the Clinical Evaluation of Language Fundamentals-Revised (Semel et al., 1987).

c PIQ measures: age 7 – Raven's Coloured Progressive Matrices (Raven, 1986); age 11 – Block Design and Picture Completion of the Wechsler Intelligence Scale for Children – Third Edition (Wechsler, 1992).

Although all the children had been identified as having significant language problems on entry to the language units, their language profiles were heterogeneous and susceptible to changes over the course of the longitudinal study. At each stage of the study, participants could be classified into four groups based on their concurrent language and PIQ scores:

- SLI – PIQ standard score 85 or above (i.e. in the normal range) and concurrent receptive or expressive language standard scores below 85.
- Non-specific language impaired – PIQ standard scores below 85 and receptive or expressive language standard scores below 85.
- Low cognition, resolved language – PIQ standard scores below 85 and receptive and expressive language standard scores 85 or above.
- Resolved language - PIQ, receptive and expressive language standard scores all 85 or above.

Participants’ language status defined under these criteria at ages 7, 8, 11 and 16 are shown in Table S1 in the online supplement. At age 7, 59.6% of the children were classified as SLI and another 10.8% were identified to have nonspecific language impairment. One child was found to have resolved language but low cognition and the remaining children (28.9%) were found to have resolved language in the presence of normal PIQ. The majority, 70.5%, of the children therefore had impaired language ability at age 7. There was little change in the language status at age 8. At ages 11 and 16, the percentages of children with SLI fell to 39.6% and 36.1%, respectively, while the percentages with nonspecific language impairment rose to 43.8% and 48.9%, respectively. The changing SLI profiles of some of the participants were thus due to their PIQ scores having fallen since they were recruited to the study. There is evidence suggesting that children with low PIQ and language skills perform in important ways much like children with a history of SLI who have PIQ within the normal range (Leonard, 2003). At ages 11 and 16, around 85% of the children showed language ability in the impaired range. For simplicity, participants are referred to as children with a history of SLI throughout the article.

#### Subgroup of children with autistic symptomatology

None of the children in the initial cohort at age 7 had a diagnosis of autism.. Two diagnostic measures were administered at 14 years of age to assess autistic symptomatology:

#### The Autism Diagnosis Interview – Revised (ADI-R)

The ADI-R is a standardized, semistructured, investigator-based interview for caregivers of individuals with possible pervasive developmental disorders, including autism (Lord, Rutter & Le Couteur, 1994). Three key areas are investigated: reciprocal social interaction, communication, and repetitive behaviours and stereotyped patterns. Each item is scored for current behaviour, with the exception of a few that are only relevant for a particular age period. Items inquiring about the lack of a behaviour or skill associated with normal development are coded for their prevalence between the ages of 4 and 5 years, in addition to the current situation. A diagnosis of autism is made if scores for each of the three areas are above the cut-offs used for the algorithm, and the individual was assessed to display some abnormality in at least one area by 36 months of age.

#### Autism Diagnostic Observation Schedule (ADOS)

The ADOS is a semistructured, standardized assessment which allows an examiner to observe behaviours that have been identified as important to the diagnosis of autism spectrum disorders (Lord, Rutter, DiLavore & Risi 1999). The ADOS thus consists of codings made from a single observation and does not include information about history. Four areas were scored: communication, social interaction, imagination, and stereotyped behaviours and restricted interests.

As noted earlier, participants for follow-up stages of the study were retained on the basis of traceability and geographical accessibility. As a result, a total of 113 children participated at age 14. For the administration of tests for autistic symptomatology, the children were further selected based on longitudinal data which showed that they met criteria for SLI at least at one time point (7, 8, 11 or 14 years). Of the 171 children in this study, 81 were administered both the ADI-R and ADOS. Of these 81 children, 53 (65.4%) did not meet the criteria for autism in either of the two diagnostic measures or the criteria for autism spectrum disorder (ASD) according to the ADOS; 28 (34.6%) met either the ADOS thresholds for autism or ASD, or the ADI-R threshold for autism. These 28 children are referred to as having a history of SLI and autistic symptomatology.

### Measures of peer relations

Two measures were used to assess peer relations: the peer problem subscale of the teacher-report version of the Strengths and Difficulties Questionnaire (SDQ) (Goodman, 1997), and teachers’ responses to three items in the Rutter Children's Behaviour Questionnaire (Rutter, 1967). The peer problem subscale of the SDQ was administered at ages 11 and 16. The SDQ is a 25 items behavioural questionnaire that can be administered to teachers, parents and children aged 11 years or over. The 25 items are divided between 5 subscales of 5 items each, with each item being coded as ‘not true’, ‘somewhat true’ or ‘certainly true’. Items of the peer problem subscale include: ‘Rather solitary, tends to play alone’, ‘Has at least one good friend’, ‘Generally liked by other children’, ‘Picked on or bullied by other children’ and ‘Gets on better with adults than with other children’. Total scores on the subscale range from 0 to 10, with higher scores indicating poorer peer relations. Scores can also be classified as ‘normal’ (0–3), ‘borderline’ (4) and ‘abnormal’ (5–10).

The Rutter Children's Behaviour Questionnaire was completed by the participants’ teachers at ages 7, 8 and 11 years. This consists of 26 statements and the teacher is asked to score each item as ‘doesn't apply’, ‘applies somewhat’ or ‘certainly applies’. Scores derived from the Rutter questionnaire and from the SDQ have been found to be highly correlated and have equivalent predictive validity (Goodman, 1997). Unlike the SDQ, there is no peer problem subscale in the Rutter questionnaire. To derive a peer problem score using the latter, ordinal logistic regression analysis was conducted to investigate which Rutter items can significantly predict the SDQ peer problem subscales at age 11, i.e. the time point when both tests were administered. The Rutter statements: ‘Not much liked by other children’, ‘Tends to do things on his own – rather solitary’ and ‘Bullies other children’, were all significant predictors, *p*s ≤ .028. To derive a peer problem score for ages 7 and 8, ratings for the three items at each age were summed. Using this method, scores could range between 0 and 6, with higher scores indicating poorer peer relations, as with the SDQ. Similarly, a Rutter-based peer problem score was also derived for age 11, giving two measures of peer relations at that age, which were highly correlated, *r* = 0.82, *p* < .001.

### Additional measures

#### Prosocial behaviour and behavioural difficulties

Strengths and Difficulties Questionnaire behavioural measures (teacher-reported version) (Goodman, 1997): prosocial behaviour, emotional symptoms, conduct problems and hyperactivity were obtained at age 11 years.. Each of these subscales ranges from 0 to 10. The prosocial subscale consists of five positive items: ‘considerate of other people's feelings’, ‘shares readily with other children’, ‘helpful if someone is hurt, upset or feeling ill’, ‘kind to younger children’ and ‘often volunteers to help others’. For this scale, the higher the rating, the more prosocial the individual. Normal prosocial behaviour is indicated by a score of between 6 and 10, whilst a score of 5 is classified as borderline. Scores between 0 and 4 are considered as displaying abnormal prosocial behaviour. In contrast, higher ratings in the emotional symptoms, conduct problems and hyperactivity subscales, are associated with increased difficulties in these areas. Examples of items constituting these latter three behavioural difficulty subscales include: ‘Many worries, often seems worried’ (emotional symptoms); ‘Often has temper tantrums or hot tempers’ (conduct problems); ‘Constantly fidgeting or squirming’ (hyperactivity). A total difficulties score can be calculated by summing the peer problem, emotional symptom, conduct problem and hyperactivity subscales, and ranges from 0 to 40, with higher ratings being associated with greater overall difficulties. As with the peer problem and prosocial subscales, thresholds for identifying normal, borderline and abnormal behaviour are also available for the other three subscales and the total difficulties score.

#### Performance IQ (PIQ)

Raven's Coloured Progressive Matrices was used to assess participants’ PIQ at age 7 (Raven, 1986). At age 11, Block Design and Picture Completion of the UK version of the Wechsler Intelligence Scale for Children – Third Edition (Wechsler, 1992) was administered.

#### Receptive and expressive language

At ages 7 and 11, receptive language was assessed using the Test for Reception of Grammar (Bishop, 1982). Expressive language at age 7 was assessed using the Bus Story Test (Renfrew, 1991) and at age 11, it was measured by the Recalling Sentences subtest of the Clinical Evaluation of Language Fundamentals-Revised (Semel, Wiig & Secord 1987).

#### Reading accuracy and comprehension

The Word Reading subtest of the British Abilities Scale (Elliot, 1983) was used to assess reading accuracy at age 7. At age 11, the Basic Reading and the Reading Comprehension subtests of the Wechsler Objective Reading Dimensions (Wechsler, 1993) were used to measure reading accuracy and reading comprehension, respectively.

#### Pragmatic language

For the first wave of the study at age 7, which was undertaken in the second half of the 1990s, teachers and speech therapists were asked about the types of language difficulties the child had, including whether they thought the child had semantic/pragmatic difficulties (illustrations of what the term meant were provided); the answers were coded as ‘yes/no’. Given the more recent changes in terminology and for ease of reading, we use the term pragmatic language impairment (PLI) when discussing this measure. Of the 171 children in this study, 54 (31.6%) were considered to have such difficulties at age 7. Pragmatic language skills were formally assessed at age 11 using the Children's Communication Checklist (CCC; Bishop, 1998). The checklist has been shown to differentiate between children with pragmatic language impairment and those with more typical SLI. Teachers or speech-language pathologists complete the checklist about the child based on good knowledge of the individual of at least 3 months. It consists of nine subscales of communication and interactive behaviour: speech, syntax, inappropriate initiation, coherence, stereotyped conversation, context, rapport, social behaviour and interests. Each scale consists of a number of behavioural items that professionals are asked to rate as ‘does not apply’, ‘applies somewhat’ or ‘definitely applies’. A composite ‘pragmatic impairment score’ is derived from the middle five scales (inappropriate initiation, coherence, stereotyped conversation, context and rapport), and a score of 132 or below is used as a cut-off for PLI. Of the 142 children included in this analysis at age 11, 33 (23.2%) met the criteria for PLI according to the CCC.

### Statistical analyses

All statistical analyses were conducted within Stata/SE 12.0 (StataCorp, 2011). Due to the skewness of the peer problem score distribution, with many children scoring 0, the peer problem scores were analysed using poisson regression. The ‘gllamm’ (generalized linear latent and mixed models; \http://www.gllamm.org; Rabe-Hesketh, Skrondal & Pickles 2004) procedure command was used to model the changes in scores across time, identifying groups (i.e. latent classes) of children with similar patterns (Nagin & Odgers 2010; Pickles & Davies 1985). The scores were modelled using a mixed poisson regression with the mean score allowed to vary on the basis of the intercept (relating to the overall level/severity of the peer problem), linear trends (allowing for differences in linear trajectory) and quadratic trends (allowing for differences in curvilinear trajectory). The models were run with an increasing number of groups with each having a different intercept and linear trend. In addition, to allow for the use of different measures earlier and later in the study, the models included a dummy variable for measure in the fixed (mean) part of the model and a factor loading in the random part (scaling the random intercept and trend). The model is thus a discrete class factor growth curve model for an overdispersed count.

We selected the model used for further analyses using both statistical goodness-of-fit criteria and interpretability, the latter taking into account the size of the groups and whether they captured forms of heterogeneity of clinical interest. The Akaike information criterion (AIC) and Bayesian information criterion (BIC), which penalizes more complex models, were used to assess the model fit. The most parsimonious model was the one with the lowest criterion value (Pickles & Croudace 2010). The chosen model was then used to calculate for each participant the empirical Bayes’ estimates for the posterior probability of belonging to each group, and each participant was assigned to the group with the highest posterior probability. Multinomial logistic regression analysis was conducted to investigate which factors predicted peer group membership. The multinomial logistic regression model is an extension of the binary logistic regression model and is used when the outcome is a multinomial variable. The model simultaneously uses all pairs of categories in the outcome variable by specifying the odds of the outcome being in one category versus another – the reference – category, with the choice of the latter being arbitrary (Agresti & Finlay 2009). For an outcome variable with four response categories, for example, we can estimate the odds of the response falling in category 4 compared with the odds of falling in category 1 (the reference category), and similarly the odds of category 3 versus category 1, category 2 versus category 1, category 4 versus category 3, etc. These results were presented as odds ratios in our study. Following each multinomial logistic regression analysis, which only tells us the relationships between the predictor(s) and pairs of outcome categories, a Wald test was conducted to examine whether the overall effect of each predictor was significant in the model. These results were presented as chi-square, *χ*^2^, statistics.

SLI is a disorder with a greater male prevalence. Hence, we also investigated the association between gender and the trajectories of peer difficulties (Conti-Ramsden & Botting 1999). All reported *p*-values are two-tailed.

## Results

The results are presented in four main sections. First, we report the pattern of overall peer problems across time. Second, we describe how different trajectory groups were identified. Third, we investigate which factors can predict trajectory group membership. In the fourth section we report the results of three subgroup analyses: children with no PLI at age 7, children with PLI at 7, and children with data on autistic symptomatology.

### Overall peer problems across time

Age trends in peer problem scores, derived from the Rutter questionnaire (ages 7, 8 and 11) and from the SDQ (ages 11 and 16), were examined within repeated measures poisson regression models (allowing for random intercept and slopes) for the two measures separately. Overall, there was a highly significant linear trend from 7 to 11, *p* < .001. There was a nonsignificant change, *p* = .14, in the mean Rutter score from 0.90 (SD = 1.05) at age 7 to 1.10 (SD = 1.19) at age 8 and a significant rise, *p* = .019, to the age-11 mean of 1.43 (SD = 1.34). Between ages 11 and 16, the mean SDQ peer problem scores increased from 2.73 (SD = 2.28) to 2.94 (SD = 2.40), but this difference was not significant, *p* = .72.

### Identifying different trajectory groups

A series of models were run allowing for 2, 3, 4 and 5 groups with different trajectories of peer problem scores. The model fit statistics are presented in Table 2. The 4-group model was the most parsimonious, offering the best fit to the data and partitioning the sample into groups with: low-level/no problems in peer relations (22.2%), childhood-limited problems (12.3%), childhood-onset persistent problems (39.2%) and adolescent-onset problems (26.3%). The average posterior probabilities for those assigned to the groups were 0.74, 0.71, 0.86 and 0.74, respectively. In all models the quadratic trends and item scaling parameters were significant. There were no significant gender differences by group, *χ*^2^(3) = 1.53, *p* = .68, by maternal education, Fisher's exact *p* = .19, or by household income at 16, Fisher's exact *p* = .30.

**Table 2:** Model fit statistics and the number and percentages of children assigned to each group

Number of groups	AIC	Sample size corrected AIC	BIC	Number (%) of individuals				
				1	2	3	4	5
2	2400.57	2401.46	2425.71	104 (60.8%)	67 (39.2%)			
3	2389.24	2390.90	2423.80	80 (46.8%)	45 (26.3%)	46 (26.9%)		
4	2376.76	2379.45	2420.74	67 (39.2%)	45 (26.3%)	21 (12.3%)	38 (22.2%)	
5	2375.52	2379.52	2428.93	60 (35.1%)	32 (18.7%)	26 (15.2%)	37 (21.6%)	16 (9.4%)

*N* = 171. AIC, Akaike information criterion; BIC, Bayesian information criterion.

**Figure 1:**
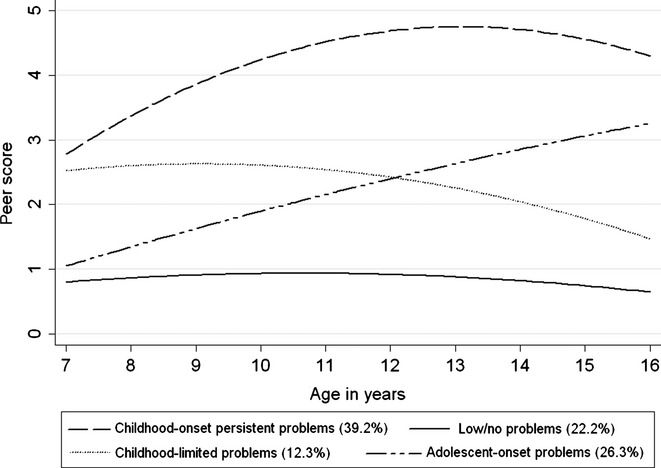
Predicted peer problem scores on the SDQ scale by peer problem groups

The fitted mean trajectories and group profiles are shown in Figure 1 and Table 3, respectively. For easier interpretation (Figure 1), we have rescaled the predicted scores derived from the Rutter to the SDQ scale. As can be seen, the group with low/no problems exhibited few problems from childhood to adolescence. For the group with childhood-limited problems, by age 16, the SDQ mean peer problem score had fallen and none of these children were classified as having borderline or abnormal levels of difficulties. The childhood-onset persistent problems group showed an increase in the Rutter mean scores between ages 7 and 11. Overall, 74.1% of children in this group at age 11 and 62.2% at age 16 were classified as having borderline or abnormal levels of peer problems. For the group with adolescent-onset problems, the percentages scoring above the SDQ threshold for borderline or abnormal levels of peer problems increased from 10% to 40.6% during this period.

**Table 3:** Means (SD) and percentages by peer problem groups

	Groups			
	Low/no problems	Childhood-limited problems	Childhood-onset persistent problems	Adolescent-onset problems
Rutter peer problem scores at 7^a^	0.4 (0.6)	1.5 (0.9)	1.4 (1.2)	0.2 (0.5)
Rutter peer problem scores at 8^a^	0.4 (0.8)	1.8 (1.1)	1.9 (1.2)	0.3 (0.5)
Rutter peer problem scores at 11^a^	0.1 (0.3)	1.1 (0.8)	2.7 (1.1)	1.0 (0.9)
SDQ peer problem scores at 11^a^	0.5 (0.7)	2.0 (0.9)	4.8 (2.0)	2.1 (1.3)
SDQ peer problem scores at 16^a^	0.4 (0.6)	0.8 (1.0)	4.6 (2.2)	3.4 (1.6)
Expressive language standard score at 7^b^	81.6 (10.1)	80.7 (8.2)	84.3 (10.6)	83.9 (9.6)
Expressive language standard score at 11^b^	71.8 (8.2)	70.8 (10.4)	73.7 (11.7)	76.4 (14.2)
Receptive language standard score at 7^c^	84.6 (9.0)	81.6 (10.8)	83.3 (12.3)	84.4 (11.7)
Receptive language standard score at 11^c^	87.4 (13.5)	82.1 (15.2)	86.0 (16.0)	88.9 (16.3)
PIQ standard score at 7^d^	104.9 (15.0)	106.9 (15.3)	104.7 (15.8)	107.1 (13.7)
PIQ standard score at 11^d^	88.7 (22.4)	86.6 (19.4)	84.1 (25.9)	84.9 (22.9)
Word reading accuracy standard score at 7^e^	84.7 (9.6)	84.1 (12.8)	87.1 (13.1)	84.7 (11.9)
Word reading accuracy standard score at 11^e^	80.5 (11.5)	81.1 (14.2)	82.8 (17.9)	79.9 (13.3)
Reading comprehension standard score at 11^e^	76.4 (14.6)	76.2 (13.2)	74.0 (15.2)	76.4 (14.0)
% with PLI at 7^f^	21.1	33.3	40.3	26.7
Pragmatic language composite score at 11^f^	147.3 (9.1)	140.8 (12.1)	134.8 (13.5)	143.7 (10.2)
% with PLI at 11^f^	6.7	20.0	38.2	16.2
SDQ prosocial scale at 11^g^	8.6 (1.8)	6.0 (2.2)	4.8 (2.6)	6.3 (2.4)
SDQ hyperactivity scale at 11^g^	2.8 (2.0)	4.5 (1.6)	4.3 (2.8)	4.0 (2.6)
SDQ emotional scale at 11^g^	1.6 (1.9)	2.4 (2.7)	3.3 (2.3)	2.8 (1.6)
SDQ conduct scale at 11^g^	0.4 (0.7)	1.7 (2.3)	2.1 (2.4)	1.1 (1.7)
SDQ total difficulties score at 11^g^	5.3 (3.0)	10.5 (4.1)	14.4 (6.1)	10.0 (4.8)
% in mainstream school without support at 11	23.7	9.5	9.1	18.2
% in mainstream school with support at 11	39.5	14.3	33.3	45.5
% in language unit/school at 11	31.6	42.9	34.9	22.7
% in other special unit/school at 11	5.3	33.3	22.7	13.6
% with autistic symptomatology at 14^h^	20.0	18.2	51.4	22.2

a Range of Rutter peer problem raw scores: 0–6; range of SDQ peer problem raw scores: 0–10.

b Expressive language measures: age 7 – Bus Story Test (Renfrew, 1991); age 11 – Recalling Sentences subtest of the Clinical Evaluation of Language Fundamentals-Revised (Semel et al., 1987).

c Receptive language measures at ages 7 and 11: Test for Reception of Grammar (Bishop, 1982).

d PIQ measures: age 7 – Raven's Coloured Progressive Matrices (Raven, 1986); age 11 – Block Design and Picture Completion of the Wechsler Intelligence Scale for Children – Third Edition (Wechsler, 1992).

e Word reading accuracy at age 7 – Word Reading subtest of the British Abilities Scale (Elliot, 1983); Word reading accuracy and reading comprehension at age 11 - Basic Reading and the Reading Comprehension subtests of the Wechsler Objective Reading Dimensions (Wechsler, 1993).

f At age 7, teachers were asked whether they thought the child had semantic/pragmatic difficulties; at age 11, PLI was assessed using the Children's Communication Checklist (CCC, Bishop, 1998). The CCC composite scores could range from 86 to 162 with a score of 132 or below indicating the presence of PLI.

g Range of SDQ raw subscale scores: 0–10; range of SDQ raw total scores: 0–40.

h Of the 171 children included in the study, 81 were tested for the presence/absence of autistic symptomatology at age 14 using both the ADOS and the ADI-R.

Table 3 also shows that 40.3% of children with persistent peer problems had PLI at age 7 compared with 21.1% of those with low/no problems, although the differences in prevalence between the four peer groups were not significant overall, *χ*^2^(3) = 4.84, *p* = .18. At age 11, these percentages were 38.2% and 6.7%, respectively, and there is a significant difference in the prevalence of PLI between the four groups, *χ*^2^(3) = 12.6, *p* = .005. Regardless of structural language status, children who showed signs of PLI are more likely than those with no such difficulties to have persistent problems with peers. In addition, the prevalence of children with autistic symptomatology was particularly high amongst those with childhood-onset persistent problems, at 51.4%. The distribution of children with autistic symptomatology across the four trajectory groups was significant, Fisher's exact *p* = .046.

### What factors predict trajectory group membership?

For those predictors where data were available at age 7 and 11 years, analyses were carried out at both ages. Each predictor was examined separately first using univariate multinomial logistic regression analysis. The results from multinomial logistic regression models, which examined the relationship between the predictor(s) and pairs of outcome categories, are presented as odds ratios. After running each multinomial regression model, a Wald test was conducted to examine whether the overall effect of each predictor was significant. These results are presented as chi-square, *χ*^2^, statistics.

Results of the univariate multinomial logistic regression models showed that receptive language, expressive language and PIQ were not significant predictors when comparing pairs of trajectory group membership, neither at age 7 nor at age 11. Wald tests also showed that these measures were not significant predictors of overall group membership, *p*s = .21–.82. Similarly, reading accuracy at age 7 and 11 and reading comprehension at 11 were not significant predictors, *p*s = .65–.95. The binary indicator for PLI at age 7 was not a significant predictor, *χ*^2^(3) = 4.74, *p* = .19. However, for children with persistent peer problems the odds of being rated by their teachers as having pragmatic language difficulties was 2.5 times higher than for children with low/no problems, OR = 2.53, 95% CI [1.01, 6.35], *p* = .048. Placement type at 11 was also not an overall significant predictor of peer group membership, *χ*^2^(9) = 16.1, *p* = .065, but children with persistent or childhood-limited problems were more likely than those with low/no problems to come from other special units/schools than from mainstream schools without support, OR = 11.3, 95% CI [1.86, 68.1], *p* = .008, and OR = 15.8, 95% CI [1.75, 141.4], *p* = .014, respectively.

Univariate analyses revealed that there were five predictors which have an overall significant effect in predicting trajectory group membership. These were: measures of pragmatic language skills at age 11, *χ*^2^(3) = 18.9, *p* < .001, prosocial behaviour, *χ*^2^(3) = 31.1, *p* < .001, hyperactivity levels, *χ*^2^(3) = 9.0, *p* = .029, and levels of emotional and conduct problems, *χ*^2^(3) = 11.7, *p* = .008, and *χ*^2^(3) = 13.8, *p* = .003.

These five variables (pragmatic language and the four SDQ subscales, all at 11 years) were subsequently entered together into a multinomial logistic regression model (Table 4). The results from this multivariate model showed that children in the childhood-onset persistent problems group and those with adolescent-onset problems were less prosocial than those with low/no peer problems, OR = 0.44, 95% CI [0.30, 0.64], *p* < .001, and OR = 0.51, 95% CI [0.36, 0.73], *p* < .001, respectively. They also showed higher levels of emotional symptoms than those with low/no problems, OR = 1.59, 95% CI [1.13, 2.24], *p* = .008, and OR = 1.46, 95% CI [1.05, 2.05], *p* = .026, respectively. Children with childhood-limited problems were also less prosocial than those with low/no problems, OR =0.53, 95% CI [0.36, 0.77], *p* = .001, but no differences in the other areas of functioning tested were found between these two groups. Children with adolescent-onset peer problems showed better pragmatic language abilities than those with childhood-onset persistent problems, OR = 1.05, 95% CI [1.01, 1.10], *p* = .020. Wald tests, which examined the overall effect of each of the five variables in this multivariate model, revealed that prosocial score at age 11 was the only variable which showed an overall significant effect in predicting group membership, *χ*^2^(3) = 19.2, *p* < .001, while emotional symptoms was of borderline significance, *χ*^2^(3) = 7.71, *p* = .053.

**Table 4:** Odds ratios (and 95% CI) from multinomial logistic regression analyses investigating factors distinguishing the differences between the peer problem groups

Variables	Childhood-onset persistent problems versus Low/no problems (reference)	Childhood-limited problems versus Low/no problems (reference)	Adolescent-onset problems versus Low/no problems (reference)
Pragmatic language at 11^a^	0.97 [0.91, 1.04], *p* = .39	1.00 [0.93, 1.07], *p* = .96	1.03 [0.96, 1.10], *p* = .46
SDQ prosocial scale at 11^b^	0.44 [0.30, 0.64], *p* < .001	0.53 [0.36, 0.77], *p* = .001	0.51 [0.36, 0.73], *p* < .001
SDQ hyperactivity scale at 11^b^	0.96 [0.69, 1.33], *p* = .80	1.10 [0.78, 1.56], *p* = .59	1.09 [0.79, 1.50], *p* = .61
SDQ emotional scale at 11^b^	1.59 [1.13, 2.24], *p* = .008	1.32 [0.91, 1.91], *p* = .15	1.46 [1.05, 2.05], *p* = .026
SDQ conduct scale at 11^b^	1.81 [0.93, 3.54], *p* = .081	1.63 [0.83, 3.22], *p* = .16	1.39 [0.71, 2.71], *p* = .34

	Adolescent-onset problems versus childhood-onset persistent problems (reference)	Childhood-limited problems versus childhood-onset persistent problems (reference)	Childhood-limited problems versus adolescent-onset problems (reference)
Pragmatic language at 11	1.05 [1.01, 1.10], *p* = .020	1.03 [0.98, 1.08], *p* = .28	0.97 [0.92, 1.03], *p* = .34
SDQ prosocial scale at 11	1.16 [0.94, 1.42], *p* = .17	1.20 [0.95, 1.52], *p* = .13	1.04 [0.81, 1.34], *p* = .76
SDQ hyperactivity scale at 11	1.14 [0.91, 1.41], *p* = .25	1.15 [0.89, 1.48], *p* = .28	1.01 [0.78, 1.32], *p* = .92
SDQ emotional scale at 11	0.92 [0.74, 1.15], *p* = .46	0.83 [0.63, 1.08], *p* = .17	0.90 [0.68, 1.19], *p* = .47
SDQ conduct scale at 11	0.76 [0.57, 1.02], *p* = .066	0.90 [0.67, 1.20], *p* = .48	1.18 [0.84, 1.64], *p* = .34

*N* = 138.

a Composite scores of the Children's Communication Checklist (CCC, Bishop, 1998) used.

b Raw scores of the SDQ used.

### Analyses by subgroup

In this section, we report three separate subgroup analyses: children with no PLI at 7 years (*n* = 117), children with PLI at 7 years (*n* = 54) and analysis with a subgroup of individuals who had data on autistic symptomatology (*n* = 81). All tables (Tables S2–S7) and figures (Figures S1–S3) for the subgroup analyses can be found in the online supplement.

#### Children with no pragmatic language difficulties at age 7

Balancing between model fit statistics (Table S2) and interpretability, the 3-class model offered the best fit to the data, partitioning the 117 children who showed no pragmatic language difficulties at age 7 into groups with: low/no peer problems (40.2%), childhood-limited problems (9.4%) and childhood-onset persistent problems (50.4%). The fitted mean trajectories (on the SDQ scale) and group profiles are shown in Figure S1 and Table S3, respectively. Table S3 also shows that, although none of these children showed pragmatic language difficulties at age 7 (based on teachers’ opinions), 12.6% met the CCC's threshold for PLI at 11.

Multinomial logistic regression was used to examine which variables could significantly predict peer group membership. Each predictor was examined separately first. The subsequent Wald test conducted for each variable showed that the overall effects of pragmatic language skills at age 11, *χ*^2^(2) = 9.04, *p* = .011, and of the SDQ subscales at 11: prosocial behaviour, *χ*^2^(2) = 17.0, *p* < .001, hyperactivity levels, *χ*^2^(2) = 6.23, *p* = .044 and levels of emotional and conduct problems, *χ*^2^(2) = 6.19, *p* = .045, and *χ*^2^(2) = 9.21, *p* = .010, were significant in predicting group membership. In addition, expressive language at 11 and reading comprehension at 11 (both using raw scores) were marginally significant, *χ*^2^(2) = 5.75, *p* = .056 and *χ*^2^(2) = 5.69, *p* = .058, respectively. For children who showed no PLI at age 7, those with childhood-onset persistent peer problems had even poorer expressive language at 11 than those with low/no peer problems, OR = 0.97, 95% CI [0.94, 1.00], *p* = .038, and those with childhood-limited problems showed better reading comprehension at 11 than those with persistent problems, OR = 1.13, 95% CI [1.00, 1.27], *p* = .044. Neither gender, household income, maternal education, placement type at 11, expressive language at 7, receptive language, reading accuracy, nor PIQ (all at 7 and 11), could contribute significantly to predicting group membership.

Following univariate analyses, pragmatic language at 11 years and the four SDQ subscales at 11 were entered together into a multivariate multinomial logistic regression model. The results (Table S4) showed that those with childhood-onset persistent problems were significantly less prosocial and showed marginally poorer pragmatic language skills than those with low/no problems, OR = 0.65, 95% CI [0.49, 0.85], *p* = .001, and OR = 0.94, 95% CI [0.89, 1.00], *p* = .057. Subsequent Wald tests revealed that of the five predictors, prosocial scores at 11 was the only variable with an overall significant effect in predicting group membership, *χ*^2^(2) = 10.1, *p* = .006. The finding was unchanged when expressive language and reading comprehension at 11, which were of marginal significance in the univariate multinomial logistic regression analyses, were additionally included into the multivariate model with the other five predictors.

Although the 3-class model offered the best fit to our data when investigating this subgroup of children with no PLI at age 7, for comparison with the main analysis, we also investigated further the 4-class model. The 117 children were partitioned into groups with: low/no problems (35.9%), childhood-limited problems (12.0%), childhood-onset persistent problems (28.2%) and adolescent-onset problems (23.9%). These results showed that a substantial proportion of children who showed no PLI at 7 still had poor peer relations.

#### Children with pragmatic language difficulties at age 7

The 54 children who showed pragmatic language difficulties at age 7 could be partitioned into two groups based on their trajectories of peer relations: low-level problems (44.4%) and persistent problems (55.6%) (see Table S5 for model fit statistics and Figure S2 and Table S6 for group profiles). In total, 44.7% of these children who showed pragmatic language difficulties at age 7 were diagnosed with PLI at age 11 according to criteria of the CCC (Table S6), compared with 12.6% of those who showed no pragmatic language problems at 7 (Table S3). Multinomial logistic regression and subsequent Wald tests revealed that, when each variable was tested individually, pragmatic language, prosocial scales and expressive language raw scores (all at age 11) were significant predictors of peer group membership, *χ*^2^(1) = 4.84, *p* = .028, *χ*^2^(1) = 7.84, *p* = .005, and *χ*^2^(1) = 4.80, *p* = .029, respectively. Children with persistent peer problems were less prosocial than those with low-level problems, OR = 0.68, 95% CI [0.51, 0.89], *p* = .005, and had poorer pragmatic language skills, OR = 0.95, 95% CI [0.90, 0.99], *p* = .028. Although expressive language scores for both groups of children at age 11 were substantially below the population mean (Table S6), children with persistent peer problems showed better expressive language skills at this age than those with low-level problems, OR = 1.04, 95% CI [1.00, 1.09], *p* = .028. These three variables were then entered together into a multivariate model (Table S7), and subsequent Wald tests revealed that expressive language at 11 was the only variable showing an overall significant effect in the prediction of peer group membership, *χ*^2^(1) = 5.39, *p* = .020.

#### Children with and without autistic symptomatology

Of the 171 children with a history of SLI in the study, 81 (47..4%) were assessed for autistic symptomatology at age 14 using both the ADI-R and ADOS; 28 of these children were found to have autistic symptomatology. Multinomial logistic regression analysis and the subsequent Wald test showed that the binary variable representing the presence/absence of autistic symptomatology was a significant predictor of peer trajectory group membership when tested on its own, *χ*^2^(3) = 8.08, *p* = .045. The odds of children showing autistic symptomatology in the childhood-onset persistent problem group was 4.2 times higher than that of children with low/no problems, OR = 4.22, 95% CI [1.02, 17.5], *p* = .047, and 3.7 times higher than for children with adolescent-onset problems, OR = 3.69, 95% CI [1.02, 13.3], *p* = .046. SDQ prosocial scores at 11 and pragmatic language at 11 were other two variables which could significantly predict peer group membership overall in the univariate multinomial logistic regression models for this subsample of children who had been tested for autistic symptomatology, *χ*^2^(3) = 13.6, *p* = .004, and *χ*^2^(3) = 10.7, *p* = .013, respectively, while SDQ emotional scores at 11 was of marginal significance, *χ*^2^(3) = 7.41, *p* = .060. However, when the binary autistic symptomatology indicator, prosocial scores and pragmatic language at 11 were all subsequently entered into a multivariate model, prosocial scores remained the only variable showing an overall significant effect in predicting group membership, *χ*^2^(3) = 9.72, *p* = .021. This finding was unchanged when emotional scores at 11, which was of marginal significance in the univariate model, was additionally included into the multivariate model. Individuals with autistic symptomatology had significantly lower prosocial scores than those without autistic symptomatology, *M* = 5.3, 95% CI [4.2, 6.3] versus *M* = 6.7, 95% CI [6.0, 7.5], F(1,72) = 5.67, *p* = .020. Further details of analyses with this subgroup can be found in the supplementary materials.

## Discussion

This investigation examined the developmental trajectories of peer relations in children with a history of SLI. Previous research suggests that, as a group, these children would experience increased peer problems from childhood to adolescence (Snowling, Bishop, Stothard, Chipchase & Kaplan 2006; St Clair et al., 2011). This was generally borne out. By analysing developmental trajectories, however, the present study showed that different pathways are followed by different groups.

We found four distinct developmental trajectories of peer relations. These four groups make sense from both empirical and clinical standpoints. The findings, on the one hand, offer positive messages regarding peer relations without problems for some children and of amelioration of difficulties across time in others. The groups with low/no problems or with childhood-limited difficulties comprised approximately one-third of the sample. On the other hand, we identified children who had persistent, or adolescent-onset, difficulties in peer relations from childhood to adolescence and, together, they represented two thirds of children with a history of SLI. Differences in peer relations were observed from early childhood (7 years) and were evident in adolescence, with trajectories diverging more widely as children entered middle childhood.

The examination of measures in middle childhood (11 years) clarified patterns of relationships. We found no significant associations between PIQ, expressive and receptive language abilities and different trajectories of peer relations. Importantly, however, the expressive and receptive language measures used in this study tapped structural language skills and not higher order language abilities such as verbal sequencing or making inferences. Since higher order language is particularly important in the adolescent period (Cohen, Farnia & Im-Bolter, 2013), this is an area worth addressing in future research. Pragmatic language ability (at 11 years), on the other hand, was a significant predictor. We also note that for children with persistent problems, if we examine their earlier profiles, the odds of having pragmatic language difficulties at 7 was 2.5 times higher than for those with low/no peer problems. Pragmatic language skills such as being able to ‘tune in’ to others’ perspectives and making inferences about verbally expressed actions and expectations during conversations are likely to affect how children with SLI are perceived and responded to by peers (Durkin & Conti-Ramsden, 2007). Further research could usefully examine which aspects of pragmatic language skills are more closely related to success or problems in peer relations in children with a history of SLI as this information would be useful in planning clinical interventions. Our findings also suggest that the more prosocial children with better pragmatic language skills and fewer difficulties in other areas of functioning (hyperactivity, emotional and conduct problems) had less difficulty in developing peer relations from childhood to adolescence.

When all these variables were examined together, prosocial behaviour and emotional symptoms were the factors most strongly associated with trajectory group membership. Prosocial behaviour can be considered as a strength or protective factor in SLI, particularly in light of the observation that the mean prosocial scores for each of the trajectory groups were in the normal and at worst borderline range. This is consistent with previous evidence that prosocial behaviour is strongly negatively correlated with social behavioural difficulties in SLI in middle childhood (Farmer, 2000). It has been argued elsewhere that prosocial behaviour can mitigate any effects due to difficulties in other areas, such as emotional symptoms and hyperactivity that can affect peer relations (Alderfer, Wiebe & Hartmann 2001). We also identified a risk factor for less favourable trajectories of peer relations: emotional problems. This evidence sheds some light on the well-established finding that a sizeable proportion of children and young people presenting for mental health problems have SLI (Cohen et al., 1998). The literature on emotional difficulties in SLI has been mixed, suggesting the presence of individual differences and a likely involvement of both child specific as well as environmental effects that require further investigation (Conti-Ramsden & Botting 2008; Goh & O'Kearney, 2013; Snowling et al., 2006). Future investigations should also explore the relationships observed in this study using more comprehensive socioemotional assessments, such as the Achenbach System of Empirically Based Assessment (ASEBA), which, as well as the parental report Child Behaviour Checklist (CBCL; Achenbach & Rescorla 2001), includes teacher and self-report measures, all appropriate for use with individuals aged 6–18 years.

Tentative evidence involving a smaller number of participants suggests that children with a history of SLI and autistic symptomatology are considerably more likely to have poorer developmental trajectories of peer relations than those without autistic symptomatology. The difficulty in detecting a significant difference when examining autistic symptomatology together with other potential predictors could partly be due to the loss of power associated with a reduced sample. This may also have restricted our ability to detect group by gender interactions. These are areas that could usefully be addressed in future research.

Although a comparison group of typically developing children was not available to this study, we are able to compare the present participants’ SDQ mean problem scores with SDQ normative data. These comparisons reveal that the SDQ mean peer problem scores of the children with a history of SLI in our study – 2.7 (SD = 2.3) at age 11 and 2.9 (SD = 2.4) at age 16 – were substantially higher than the SDQ norms of 1.4 (SD = 1.8) for 11–15 year olds (http://www.sdqinfo.com/norms/UKNorm3.pdf). This indicates that children with a history of SLI in our study had poorer peer relations than children in the general population of similar ages. As such, irrespective of whether the risk/resilience factors would also be the same for children without language impairment, the present results are informative and have important clinical implications for those with language impairment. Our study also relied only on teacher-reported behaviour. If parent- or peer-reported ratings were available, they may provide additional insights and different perspectives. In addition, our study uses a language unit sample which is likely to include children with severe language problems. Hence, our findings may not be generalizable to, for example, community-based samples of children with SLI who may exhibit difficulties in the more mild to moderate range. These are areas that could usefully be addressed in future research.

### Clinical implications

We provide encouraging evidence that some children with a history of SLI progress from childhood to adolescence with relatively low levels of peer problems. Nevertheless, the majority showed childhood-onset persistent or adolescent-onset difficulties. This is important information in terms of our understanding of the developmental course of peer relations in those with language impairments and has implications for identification, referral to services and the targeting of interventions.

Clinically, our findings suggest that the identification of pragmatic difficulties and emotional problems could be critical to the amelioration of potential difficulties in peer relations. In this study, the use of teacher report of pragmatic difficulties were informative at age 7 as was the CCC and the SDQ emotional scale in middle childhood, at age 11. Results of randomized controlled trials show that pragmatic difficulties (Adams et al., 2012) and emotional problems (Sanders, Baker & Turner 2012) can be addressed effectively, in at least some children. Our results also suggest that, clinically, it is advisable not only to identify deficits but to evaluate the extent to which potential protective and/or positive factors are present. We found that the SDQ prosocial scale in middle childhood to be a particularly strong predictor of differences in the trajectories of peer relations of children with a history of SLI. Prosocial behaviours are also open to contextual influence and intervention. For example, children whose parents express responsiveness and warmth are more likely to display prosocial behaviours (Zhou et al., 2002), as are adolescents whose best friends engage in such behaviours (Barry & Wentzel 2006). Interventions, such as Family Talk Intervention (Solantous, Paavonen, Toikka & Punamäki, 2010) and school-based strategies (Riedel, 2002; Solomon, Battistich, Watson, Schaps & Lewis 2000) have been shown to be effective in improving prosocial behaviours in children. To the authors’ knowledge, however, there are no intervention studies that have directly targeted prosocial behaviours in children with language impairments. Finally, it is salutary to reflect that, in most countries, provision for language therapy diminishes for children post- the elementary school range, and provision for social skills training in peer relations in these children is scant at all ages.

## Key points

- Four distinct developmental trajectories of peer relations in children with a history of SLI were identified.
- Two-thirds of children showed childhood-onset persistent or adolescent-onset problems. A third showed low-level/no or childhood-limited peer problems.
- For the 39% of children with childhood-onset persistent problems, the odds of showing pragmatic language difficulties at 7 years was 2.5 times higher than for those with low/no peer problems.
- Prosocial behaviour, the variable most strongly associated with trajectory group membership, may act as a protective factor.
- Increased emotional symptoms were associated with poor trajectories of peer relations.

## Supporting Information

Table S1 Language status of children at ages 7, 8, 11 and 16. **Table S2** Model fit statistics and the number and percentages of children assigned to each peer problem group-Children with no PLI at age 7. **Table S3** Means (SD) and percentages by peer problem groups- Children with no PLI at age 7. **Table S4** Odds ratios (and 95%CI) from multinomial logistic regression analyses-Children with no PLI at age 7. **Table S5** Model fit statistics and the number and percentages of children assigned to each peer problem group – Children with PLI at age 7. **Table S6** Means (SD) and percentages by peer problem groups – Children with PLI at age 7. **Table S7** Odds ratios (and 95% CI) from multinomial logistic regression analyses – only children who showed PLI at age 7. **Figure S1** Predicted peer problem scores on the SDQ scale by peer problem groups – Children with no PLI at age 7 (*N* = 117). **Figure S2** Predicted peer problem scores on the SDQ scale by peer problem groups – Children with PLI at age 7 (*N* = 54). **Figure S3** Breakdown of participants by PLI and autistic symptomatology (ASD) over time.
