# Supplementary material for: Longitudinal trajectories of peer relations in children with specific language impairment
Source: J Child Psychol Psychiatry. 2014 Jan 11;55(5):516–27. doi: 10.1111/jcpp.12190 (PMC4283728; doi:10.1111/jcpp.12190)
Supplement: Table S1 — Language status of children at ages 7, 8, 11 and 16. Table S2 Model fit statistics and the number and percentages of children assigned to each peer problem group-Children with no PLI at age 7. Table S3 Means (SD) and percentages by peer problem groups- Children with no PLI at age 7. Table S4 Odds ratios (and 95%CI) from multinomial logistic regression analyses-Children with no PLI at age 7. Table S5 Model fit statistics and the number and percentages of children assigned to each peer problem group – Children with PLI at age 7. Table S6 Means (SD) and percentages by peer problem groups – Children with PLI at age 7. Table S7 Odds ratios (and 95% CI) from multinomial logistic regression analyses – only children who showed PLI at age 7. Figure S1 Predicted peer problem scores on the SDQ scale by peer problem groups – Children with no PLI at age 7 (N = 117). Figure S2 Predicted peer problem scores on the SDQ scale by peer problem groups – Children with PLI at age 7 (N = 54). Figure S3 Breakdown of participants by PLI and autistic symptomatology (ASD) over time. [file jcpp0055-0516-SD1.doc]

**Supplementary online materials**

**Further demographics information of participants**

Information on maternal education collected at age 16 was available for 123 (71.9%) of the 171 children in the study. Of these 123 children, 22.8% had mothers with no formal educational qualifications. A further 62.6% had a basic level of education (GCSE O/A-levels or equivalent) and 14.6% had a university or postgraduate degree. Data on household income at age 16 were available for 124 participants, with 16.1% coming from households with an annual income of up to £10,401, 28.2% with an income of between £10,401 and £20,800, 33.1% with an income of between £20,801 and £36, 400, and 22.6% from households earning over £36,400.

**Table S1. *Language status of children at ages 7, 8, 11 and 16***

| Language status | Age 7 (%) | Age 8 (%) | Age 11 (%) | Age 16 (%) |
| --- | --- | --- | --- | --- |
| SLI | 59.6 | 60.5 | 39.6 | 36.1 |
| Non-specific language impaired | 10.8 | 10.2 | 43.8 | 48.9 |
| Low cognition, resolved language | 0.6 | 1.3 | 2.4 | 4.5 |
| Resolved language | 28.9 | 28.0 | 14.2 | 10.5 |
| Total | 100 | 100 | 100 | 100 |
|  |  |  |  |  |
| *N* ^a^ | 166 | 157 | 169 | 133 |

^a^ Number of children not including those whose language status was unidentified due to missing data.

**Table S2.** ***Model fit statistics and the number and percentages of children assigned to each peer problem group – Children with no PLI at age 7***

| Number of groups | AIC ^a^ | Sample size corrected AIC | BIC ^b^ | Number (%) of individuals | | | |  |
| --- | --- | --- | --- | --- | --- | --- | --- | --- |
|  |  |  |  | 1 | 2 | 3 | 4 |  |
| 2 | 1589.17 | 1590.50 | 1611.26 | 67 (57.3%) | 50 (42.7%) |  |  |  |
| 3 | 1581.06 | 1583.58 | 1611.45 | 59 (50.4%) | 11 (9.4%) | 47 (40.2%) |  |  |
| 4 | 1576.15 | 1580.27 | 1614.82 | 33 (28.2%) | 14 (12.0%) | 28 (23.9%) | 42 (35.9%) |  |

Note:

*N* = 117

^a^ AIC - Akaike information criterion

^b^ BIC - Bayesian information criterion

***Figure S1.* Predicted peer problem scores on the SDQ scale by peer problem groups – Children with no PLI at age 7 (*N* = 117)**

**
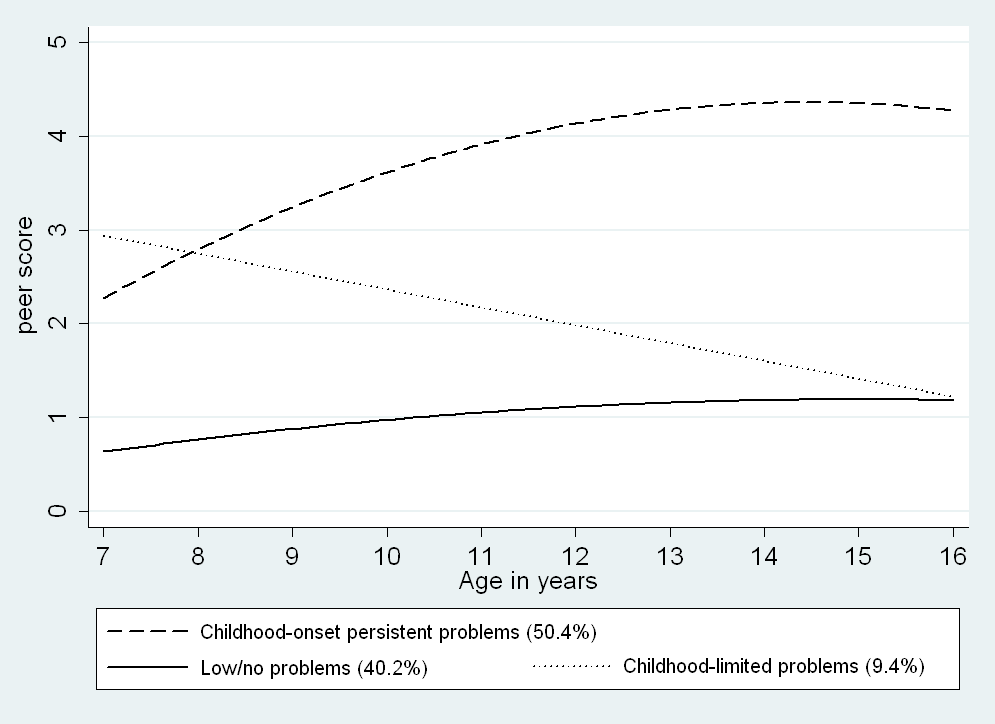
**

**Table S3. *Means (SD) and percentages by peer problem groups* *- Children with no PLI at age 7***

|  | Groups | | | |
| --- | --- | --- | --- | --- |
|  | Low/no problems | Childhood-limited problems | Childhood-onset persistent problems | All |
|  |  |  |  |  |
| Rutter peer problem scores at 7 ^a^ | 0.3 (0.5) | 1.6 (1.0) | 1.0 (1.2) | 0.8 (1.0) |
| Rutter peer problem scores at 8 ^a^ | 0.1 (0.3) | 1.8 (1.0) | 1.4 (1.1) | 0.9 (1.1) |
| Rutter peer problem scores at 11 ^a^ | 0.2 (0.4) | 1.3 (1.0) | 2.2 (1.2) | 1.3 (1.3) |
| SDQ peer problem scores at 11 ^a^ | 0.9 (1.0) | 1.5 (1.3) | 4.0 (2.0) | 2.5 (2.2) |
| SDQ peer problem scores at 16 ^a^ | 1.3 (1.2) | 0.3 (0.7) | 4.4 (2.0) | 2.7 (2.3) |
|  |  |  |  |  |
| Expressive language standard score at 7 ^b^ | 82.8 (10.0) | 80.9 (9.3) | 84.9 (10.9) | 83.7 (10.4) |
| Expressive language standard score at 11 ^b^ | 75.2 (11.7) | 72.6 (10.4) | 71.7 (12.1) | 73.2 (11.8) |
|  |  |  |  |  |
| Receptive language standard score at 7 ^c^ | 84.4 (9.7) | 87.0 (10.8) | 83.9 (12.5) | 84.4 (11.2) |
| Receptive language standard score at 11 ^c^ | 88.5 (13.8) | 88.0 (18.2) | 84.0 (14.4) | 86.2 (14.6) |

**Table S3 (cont.) *Means (SD) and percentages by peer problem groups* *- Children with no PLI at age 7***

|  | Low/no problems | Childhood-limited problems | Childhood-onset persistent problems | All |
| --- | --- | --- | --- | --- |
| PIQ standard score at 7 ^d^ | 104.5 (14.8) | 109.0 (15.6) | 104.7 (13.9) | 105.0 (14.4) |
| PIQ standard score at 11 ^d^ | 86.7 (22.2) | 89.3 (22.0) | 80.4 (24.2) | 83.8 (23.3) |
|  |  |  |  |  |
| Word reading accuracy standard score at 7 ^e^ | 83.1 (8.7) | 86.5 (11.7) | 84.6 (10.1) | 84.2 (9.7) |
| Word reading accuracy standard score at 11 ^e^ | 79.4 (9.9) | 86.0 (15.3) | 77.9 (13.9) | 79.3 (12.7) |
| Reading comprehension standard score at 11 ^e^ | 77.3 (11.0) | 81.4 (15.2) | 72.5 (14.2) | 75.3 (13.3) |
|  |  |  |  |  |
| Pragmatic language composite score at 11 ^f^ | 147.8 (8.7) | 147.3 (11.1) | 141.0 (10.3) | 144.3 (10.3) |
| % with PLI at 11 ^f^ | 5.4 | 10.0 | 18.8 | 12.6 |

**Table S3 (cont.) *Means (SD) and percentages by peer problem groups* *- Children with no PLI at age 7***

|  | Low/no problems | Childhood-limited problems | Childhood-onset persistent problems | All |
| --- | --- | --- | --- | --- |
| SDQ prosocial scale at 11 ^g^ | 8.0 (2.3) | 6.7 (2.4) | 5.5 (2.5) | 6.6 (2.7) |
| SDQ hyperactivity scale at 11 ^g^ | 3.1 (2.3) | 3.7 (1.8) | 4.5 (2.8) | 3.8 (2.6) |
| SDQ emotional scale at 11 ^g^ | 2.0 (1.9) | 1.9 (2.8) | 3.1 (2.1) | 2.5 (2.1) |
| SDQ conduct scale at 11 ^g^ | 0.4 (0.8) | 1.4 (2.7) | 2.0 (2.6) | 1.3 (2.2) |
| SDQ total difficulties score at 11 ^g^ | 6.6 (3.6) | 8.5 (5.3) | 13.6 (6.5) | 10.4 (6.3) |
|  |  |  |  |  |
| % in mainstream school without support at 11 | 19.2 | 27.3 | 13.8 | 17.2 |
| % in mainstream school with support at 11 | 42.6 | 18.2 | 37.9 | 37.9 |
| % in language unit/school at 11 | 27.7 | 18.2 | 27.6 | 26.7 |
| % in other special unit/school at 11 | 10.6 | 36.4 | 20.7 | 18.1 |
|  |  |  |  |  |
| % with autistic symptomatology at 14 ^h^ | 20.0 | 14.3 | 31.0 | 25.0 |

Note:

^a^ Range of Rutter peer problem raw scores: 0-6; range of SDQ peer problem raw scores: 0-10**.**

**Table S3 (cont.) *Means (SD) and percentages by peer problem groups* *- Children with no PLI at age 7***

Note:

^b^ Expressive language measures: age 7 - Bus Story Test (Renfrew, 1991); age 11 - Recalling Sentences subtest of the Clinical Evaluation of Language Fundamentals-Revised (Semel, Wiig, & Secord, 1987).

^c^ Receptive language measures at ages 7 and 11: Test for Reception of Grammar (Bishop, 1982).

^d^ PIQ measures: age 7 - Raven’s Coloured Progressive Matrices (Raven, 1986); age 11 - Block Design and Picture Completion of the Wechsler Intelligence Scale for Children – Third Edition (Wechsler, 1992).

^e^ Word reading accuracy at age 7 - Word Reading subtest of the British Abilities Scale (Elliot, 1983); Word reading accuracy and reading comprehension at age 11 - Basic Reading and the Reading Comprehension subtests of the Wechsler Objective Reading Dimensions (Wechsler, 1993).

^f^ PLI was assessed using the Children’s Communication Checklist (CCC, Bishop, 1998). The CCC composite scores could range from 86 to 162 with a score of 132 or below indicating the presence of PLI.

^g^ Range of SDQ raw subscale scores: 0-10; range of SDQ raw total scores: 0-40.

^h^ Of the 117 children who showed no pragmatic language difficulties at age 7, 56 were tested for the presence/absence of autistic symptomatology at age 14 using both the ADOS and the ADI-R.

**Table S4. *Odds ratios (and 95%CI) from multinomial logistic regression analyses investigating factors distinguishing the differences between the three peer problem groups including only children who showed no PLI at age 7***

| Variables | Childhood-onset persistent problems  vs  Low/no problems (reference) | Childhood-limited problems  vs  Low/no problems (reference) | Childhood-limited problems  vs  Childhood-onset persistent problems |
| --- | --- | --- | --- |
| Pragmatic language at 11 ^a^ | 0.94 [0.89, 1.00], *p* = .057 | 1.00 [0.91, 1.09], *p* = .98 | 1.06 [0.97, 1.15], *p* = .20 |
| SDQ prosocial scale at 11 ^b^ | 0.65 [0.49, 0.85], *p* = .001 | 0.77 [0.53, 1.10], *p* = .14 | 1.18 [0.85, 1.64], *p* = .32 |
| SDQ hyperactivity scale at 11 ^b^ | 0.87 [0.66, 1.14], *p* = .31 | 0.88 [0.60, 1.28], *p* = .50 | 1.01 [0.71, 1.45], *p* = .95 |
| SDQ emotional scale at 11 ^b^ | 1.16 [0.89, 1.52], *p* = .26 | 0.96 [0.63, 1.44], *p* = .83 | 0.82 [0.55, 1.22], *p* = .33 |
| SDQ conduct scale at 11 ^b^ | 1.41 [0.95, 2.10], *p* = .087 | 1.38 [0.86, 2.21], *p* = .18 | 0.97 [0.66, 1.43], *p* = .90 |

**Table S4 (cont). *Odds ratios (and 95%CI) from multinomial logistic regression analyses investigating factors distinguishing the differences between the three peer problem groups including only children who showed no PLI at age 7***

Note:

*N* = 92

^a^ Composite scores of the Children’s Communication Checklist (CCC, Bishop, 1998) used.

^b^ Raw scores of the SDQ used.

**Table S5.** ***Model fit statistics and the number and percentages of children assigned to each peer problem group – Children with PLI at age 7***

| Number of groups | AIC ^a^ | Sample size corrected AIC | BIC ^b^ | Number (%) of individuals | | |  |
| --- | --- | --- | --- | --- | --- | --- | --- |
|  |  |  |  | 1 | 2 | 3 |  |
| 1 | 854.19 | 855.01 | 862.15 | 54 (100%) |  |  |  |
| 2 | 805.28 | 808.48 | 821.19 | 30 (55.6%) | 24 (44.4%) |  |  |
| 3 | 808.64 | 814.93 | 830.52 | 5 (9.3%) | 27 (50.0%) | 22 (40.7%) |  |

Note:

*N* = 54

^a^ AIC - Akaike information criterion

^b^ BIC - Bayesian information criterion

***Figure S2.* Predicted peer problem scores on the SDQ scale by peer problem groups – Children with PLI at age 7 (*N* = 54)**


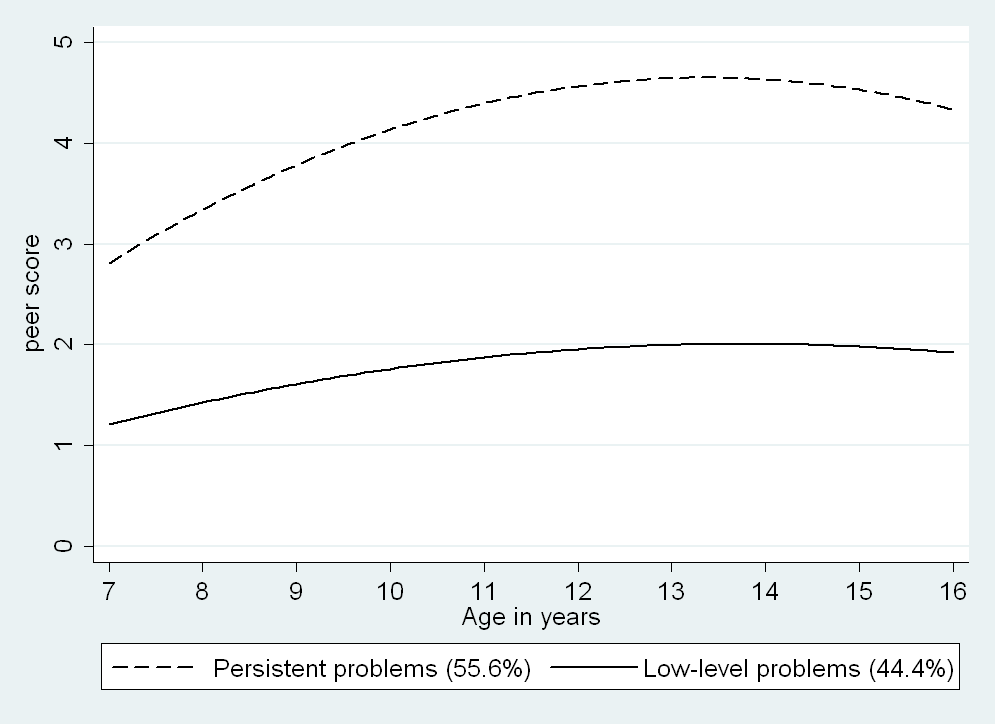


**Table S6. *Means (SD) and percentages by peer problem groups* *- Children with PLI at age 7***

|  | Groups | | |
| --- | --- | --- | --- |
|  | Low-level problems | Persistent problems | All |
|  |  |  |  |
| Rutter peer problem scores at 7 ^a^ | 0.5 (0.7) | 1.6 (1.2) | 1.1 (1.1) |
| Rutter peer problem scores at 8 ^a^ | 0.8 (0.8) | 2.2 (1.3) | 1.6 (1.3) |
| Rutter peer problem scores at 11 ^a^ | 0.7 (0.8) | 2.7 (1.0) | 1.7 (1.4) |
| SDQ peer problem scores at 11 ^a^ | 1.5 (1.3) | 4.8 (2.1) | 3.2 (2.4) |
| SDQ peer problem scores at 16 ^a^ | 1.9 (1.7) | 4.4 (2.5) | 3.5 (2.5) |
|  |  |  |  |
| Expressive language standard score at 7 ^b^ | 80.3 (9.3) | 83.6 (8.5) | 82.1 (9.0) |
| Expressive language standard score at 11 ^b^ | 71.0 (9.6) | 77.5 (12.1) | 74.5 (11.4) |
|  |  |  |  |
| Receptive language standard score at 7 ^c^ | 82.1 (11.0) | 82.2 (11.7) | 82.2 (11.3) |
| Receptive language standard score at 11 ^c^ | 84.8 (17.1) | 89.4 (17.7) | 87.3 (17.4) |

***Table S6 (cont.) Means (SD) and percentages by peer problem groups - Children with PLI*** at age 7

|  | Low-level problems | Persistent problems | All |
| --- | --- | --- | --- |
| PIQ standard score at 7 ^d^ | 107.3 (14.5) | 106.9 (17.7) | 107.1 (16.1) |
| PIQ standard score at 11 ^d^ | 90.0 (22.9) | 89.3 (24.6) | 89.7 (23.6) |
|  |  |  |  |
| Word reading accuracy standard score at 7 ^e^ | 85.1 (15.8) | 91.1 (15.0) | 88.4 (15.5) |
| Word reading accuracy standard score at 11 ^e^ | 81.1 (16.6) | 89.4 (19.4) | 85.7 (18.5) |
| Reading comprehension standard score at 11 ^e^ | 73.6 (18.8) | 77.6 (15.0) | 75.8 (16.7) |
|  |  |  |  |
| Pragmatic language composite score at 11 ^f^ | 137.7 (12.0) | 128.8 (13.8) | 133.1 (13.6) |
| % with PLI at 11 ^f^ | 30.4 | 58.3 | 44.7 |
|  |  |  |  |
| SDQ prosocial scale at 11 ^g^ | 6.5 (2.2) | 4.3 (2.5) | 5.4 (2.6) |
| SDQ hyperactivity scale at 11 ^g^ | 3.9 (2.0) | 4.2 (2.8) | 4.1 (2.4) |
| SDQ emotional scale at 11  ^g^ | 2.4 (2.1) | 3.3 (2.3) | 2.9 (2.2) |
| SDQ conduct scale at 11 ^g^ | 1.4 (1.6) | 1.6 (1.8) | 1.5 (1.7) |
| SDQ total difficulties score at 11 ^g^ | 9.2 (4.5) | 13.6 (5.1) | 11.5 (5.3) |

***Table S6 (cont.) Means (SD) and percentages by peer problem groups - Children with PLI*** at age 7

|  | Low-level problems | Persistent problems | All |
| --- | --- | --- | --- |
| % in mainstream school without support at 11 | 16.7 | 3.5 | 9.4 |
| % in mainstream school with support at 11 | 25.0 | 34.5 | 30.2 |
| % in language unit/school at 11 | 41.7 | 44.8 | 43.4 |
| % in other special unit/school at 11 | 16.7 | 17.2 | 17.0 |
|  |  |  |  |
| % with autistic symptomatology at 14 ^h^ | 25 | 70.6 | 56 |

Note:

^a^ Range of Rutter peer problem raw scores: 0-6; range of SDQ peer problem raw scores: 0-10**.**

^b^ Expressive language measures: age 7 - Bus Story Test (Renfrew, 1991); age 11 - Recalling Sentences subtest of the Clinical Evaluation of Language Fundamentals-Revised (Semel, Wiig, & Secord, 1987).

^c^ Receptive language measures at ages 7 and 11: Test for Reception of Grammar (Bishop, 1982).

^d^ PIQ measures: age 7 - Raven’s Coloured Progressive Matrices (Raven, 1986); age 11 - Block Design and Picture Completion of the Wechsler Intelligence Scale for Children – Third Edition (Wechsler, 1992).

**Table S6 (cont.) *Means (SD) and percentages by peer problem groups* *- Children with PLI at age 7***

Note:

^e^ Word reading accuracy at age 7 - Word Reading subtest of the British Abilities Scale (Elliot, 1983); Word reading accuracy and reading comprehension at age 11 - Basic Reading and the Reading Comprehension subtests of the Wechsler Objective Reading Dimensions (Wechsler, 1993).

^f^ PLI was assessed using the Children’s Communication Checklist (CCC, Bishop, 1998). The CCC composite scores could range from 86 to 162 with a score of 132 or below indicating the presence of PLI.

^g^ Range of SDQ raw subscale scores: 0-10; range of SDQ raw total scores: 0-40.

^h^ Of the 54 children who showed pragmatic language difficulties at age 7, 25 were tested for the presence/absence of autistic symptomatology at age 14 using both the ADOS and the ADI-R.

**Table S7. *Odds ratios (and 95%CI) from multinomial logistic regression analyses investigating factors distinguishing the differences between the two peer problem groups including only children who showed PLI at age 7***

| Variables | Persistent problems  vs  Low-level problems (reference) |
| --- | --- |
|  |  |
| Pragmatic language at 11 ^a^ | 0.94 [0.89, 1.01], *p* = .096 |
| SDQ prosocial scale at 11 ^b^ | 0.82 [0.59, 1.14], *p* = .24 |
| Expressive language at 11 ^c^ | 1.07 [1.01, 1.13], *p* = .020 |

Note:

*N* = 47

^a^ Composite scores of the Children’s Communication Checklist (CCC, Bishop, 1998) used.

^b^ Raw scores of the SDQ prosocial scale used.

^C^ Measure of expressive language at age 11: Recalling Sentences subtest of the Clinical Evaluation of Language Fundamentals-Revised (Semel, Wiig, & Secord, 1987). Raw scores used.

**Further analyses of subgroups of children with and without autistic symptomatology**

Since children with autistic symptomatology were more likely than those who did not have this condition to be found in the childhood-onset persistent problem group, we reran the trajectory models by excluding the 28 children who had been diagnosed with autistic symptomatology. The 4-class model again offered the best fit to the data, partitioning the 143 children with no autistic symptomatology into groups with: low-level/no problems in peer relations (25.2%), childhood-limited problems (18.9%), childhood-onset persistent problems (21.7%), and adolescent-onset problems (34.3%). The corresponding percentages when all children were included in the analysis (*N* = 171) were: 22.2%, 12.3%, 39.2%, and 26.3%, respectively. The percentage of children with persistent problems therefore fell when those with autistic symptomatology had been excluded from the analysis. The conclusion however remained largely unchanged: prosocial score at 11 and emotional symptoms at 11 were the two variables which could significantly predict peer group membership overall, χ^2^(3) = 14.0, *p* = .003, and χ^2^(3) = 8.18, *p* = .042 (emotional symptoms was of marginal significance in the analysis with all children).

**Investigating the association between autistic symptomatology and PLI at 7 years.**

Figure C presents information on the characteristics (PLI, ASD) of participants across time. Of the 117 children with no PLI at age 7, 56 were assessed for autistic symptomatology of whom 14 (25%) were found to show such condition. Of these 14 children, 9 were in the childhood-onset persistent peer problem group, 1 had childhood-limited problems and 4 showed no/low problems (Figure A). The differences were not significant, although this could partly be due to the small sample size, Fisher’s exact *p* = .62. Univariate multinomial logistic regression analysis and the subsequent Wald test also revealed that the binary indicator for autistic symptomatology was not a significant predictor of peer group membership overall, χ^2^(2) = 1.22, *p* = .54, while prosocial scores at 11 was again the only significant predictor, χ^2^(2) = 7.09, *p* = .029.

For the subgroup with PLI at 7 years (*N* = 54), 14 of the 25 children assessed for autistic symptomatology were found to have this condition, i.e. 56% of these children had autistic sympatomatology compared with 25% of those who had no signs of PLI at 7 years. Twelve of these 14 children had childhood-onset persistent peer problems and the other 2 had low-level problems (Figure B). Multinomial logistic regression analysis and Wald test revealed that the binary indicator of autistic symptomatology was a significant predictor of peer group membership, χ^2^(1) = 4.10, *p* = .043. For these children with PLI at 7, the odds of those with persistent peer problems having autistic symptomatology was 7.2 times higher than that of children with low-level problems, *OR* = 7.20, 95% CI [1.07, 48.6], *p* = .043. No other variables tested were found to be significant.

***Figure S3.* Breakdown of participants by PLI and autistic symptomatology (ASD) over time**
